# Genetic variations in S gene of porcine epidemic diarrhoea virus from 2018 in Sichuan Province, China

**DOI:** 10.1002/vms3.326

**Published:** 2020-09-04

**Authors:** Fei Li, Yubing Zeng, Rubo Zhang, Kenan Peng, Chaoyuan Jiang, Zhiwen Xu, Ling Zhu

**Affiliations:** ^1^ College of Veterinary Medicine Sichuan Agricultural University – Chengdu Campus Chengdu Sichuan China

**Keywords:** genetic evolution, genogroup, PEDV, phylogenetic analysis

## Abstract

Porcine epidemic diarrhoea virus (PEDV) belongs to the family Coronavirus, a genus of coronavirus, a highly contact‐infectious intestinal disease pathogen. In this study, we downloaded 62 PEDV S gene sequences uploaded to GenBank, including 10 uploaded by our laboratory from 2018, and constructed a PEDV S gene evolution tree using MEGA V7.0 software. Phylogenetic tree analysis indicated that the genogroup of PEDV in Sichuan Province was divided into three coexisting genogroups (GII‐a, GII‐b and GI‐a), of them, GII‐a has become the main genogroup in the province due to its prevalence and range of spread. Amino acid sequence analysis showed that there were amino acid insertions and deletions in the S protein encoded by the amplified S gene, and there were amino acid mutations in the COE and SS6 of the epitope in the amplified S protein. These results provide a basic research theory for understanding the prevalence of PEDV variation and controlling PED in Sichuan.

## Graphical abstract

In 2018, 10 strains of PEDV S gene were amplified in Sichuan. The genetic evolution analysis of 10 strains of PEDV and the reference strain showed that GII‐a was the main strain in Sichuan. Provide reference for PEDV prevention and control in Sichuan.

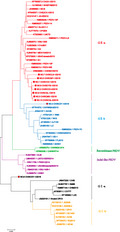

## 1 INTRODUCTION

Porcine epidemic diarrhoea virus (PEDV), the aetiological agent of Porcine epidemic diarrhoea (PED), is a large‐enveloped RNA virus. PEDV belongs to the family Coronavirus, a genus of coronavirus, which causes a highly contact‐infectious intestinal disease in pigs, the symptoms including diarrhoea, vomiting, anorexia and dehydration in piglets (Have, Moving, Svansson, Uttenthal, & Bloch, 1992; Sueyoshi et al., 1995). The PEDV genome is approximately 28 kb long with a 5’ cap and a 3’ polyadenylated tail and comprises a 5’ untranslated region (UTR), at least seven open reading frames, and a 3’ UTR. The main structural proteins include glycosylated spike (S) protein, envelope (E) protein, membrane (M) protein and nucleocapsid (N) protein (Lee, 2015). The S protein is composed of 1,383 amino acids and is the largest structural protein on the surface of virions. According to the S proteins of other coronaviruses, PEDV S proteins can be divided into S1 (amino acids 1–789) and S2 (amino acids 790–1383). The S1 region contains several virus main neutralizing epitopes and receptor binding domains, which are closely related to viral antigenicity and adsorption invasion (Sun et al., 2007). As the transmembrane part of the S protein, the S2 region is relatively stable and plays an important role in the signal transduction process of virus–host cell membrane fusion. Its fusion activity also determines the tissue tropism and cell infection ability of the virus (Kang et al., 2005).

The S protein can activate the host to produce neutralizing antibodies, and four neutralizing epitopes have been found (COE: aa 499–638, SS2: aa 748–755, SS6: aa 764–771, 2C10: aa1 368–1 374; Jun‐Wei, Di‐Qiu, Di‐Qiu, & Yi‐Jing, 2012; Stevenson et al., 2013; Yao‐Wei et al., 2013). The S gene mutates easily. Therefore, taking the S gene as the object of molecular epidemiological investigation can reflect the variation in the whole genome to a certain extent. Phylogenetic studies of the S gene suggested that PEDV can be genetically separated into two groups: genogroup 1 (GI) and genogroup 2 (GII). Each genogroup can be further divided into subgroups 1a and 1b, and 2a and 2b respectively (Lee, 2015; Pensaert & de Bouck, 1978). Therefore, the S protein plays an important role in the genetic evolution of PEDV.

Since PED was first discovered, it is still a destructive bowel disease, causing serious losses in China. In the early 1990s, a vaccine containing an inactivated prototype CV777 strain was developed and has since been widely used in the entire pig industry in China until 2010. Although PEDV infection still affects Chinese pig farms, it is sporadic and regional. A clinical outbreak of acute diarrhoea broke out in the southern provinces in October 2010 and quickly swept the country, although most pig herds were vaccinated with CV777‐inactivated or ‐attenuated vaccine (Rui‐Qin et al., 2012; Wentao et al., 2012; Xiao‐Meng et al., 2013). The mortality rate of PED in piglets soared to 80%–100%, and the pig industry suffered a devastating blow (Rui‐Qin et al., 2012). Among the more than 600 samples collected from nine provinces in China from 2010 to 2011, the positive rate of PEDV was more than 50%. The AJ1102 strains isolated from positive materials and strains CHGD‐01, BJ‐2011–1 and CH/FJND‐3/2011 isolated during this period all belonged to the GII genome. Compared with CV777, the S gene had multiple amino acid deletions and additions, and the GI and GII genomes were not genetically closely related. Sichuan Province has the largest number of pigs in China, and PED has killed large numbers of piglets on some farms in recent years. Therefore, this study investigated the latest changes in the S gene of different PEDV isolates in Sichuan to understand the main epidemic strains of PEDV in this area, so as to provide theoretical basis for the development and application of vaccines.

## 2 MATERIAL AND METHODS

### 2.1 Samples

Naturally infected pigs suspected of having PEDV were collected in various parts of Sichuan Province and the intestinal tissues and contents of the affected pigs were stored at –80°C after grinding with liquid nitrogen.

### 2.2 Primer design and synthesis

Four pairs of specific primers were designed according to the conserved regions of CV777 (GenBank:AF353511.1) published in GenBank and PEDV mutants published in recent years, and the S gene was divided into four fragments (S1, S2, S3 and S4). During amplification, there was partial overlap between each segment, the primers are synthesized by Sangon Biotech. Primer information is shown in Table 1.

**Table 1:** Sequences of amplified PEDV S gene

Name	Sequence	Annealing temperature (°C)	Amplification size (bp)
S1F	CCATTAGTGATGTTGTGTTAGG	54°C	1,489
S1R	AAGAATACGCTGAATGG		
S2F	CATGGCACTGACGATGA	52°C	1,328
S2R	CCATCACCATTAAACGAAC		
S3F	GCATGTAAGACCATAGAGTCAG	52°C	1,143
S3R	CATACGTCGCGATGAAAC		
S4F	ATTGCCTTGACTCTACGTG	54°C	722
S4R	CAACTCTTGGACAGCATC		

### 2.3 Complete genome extraction and sequencing

Samples were diluted with five volumes of 0.9% saline (w/v), frozen and thawed with liquid nitrogen three times and then clarified by centrifugation for 5 min at 3,000 rpm. Three hundred microlitres of the supernatants were used for RNA extraction using TRIzol (Total RNA Extractor)(Solarbio Co), according to the manufacturer's instructions. cDNA was synthesized by PrimeScript^TM^ RT Reagent Kit (TaKaRa Co), according to the manufacturer's instructions. The S gene fragment was amplified by 2 X High Fidelity PCR Master Mix (Sangon Biotech Co). Briefly, the reaction was carried out at 95°C for 3 min (for denaturation), 35 cycles of 95°C for 15 s (for denaturation), 52°C/54°C for 15 s (for annealing) and 72°C for 90 s (for extension), followed by 72°C for 5 min (for final extension). The products were examined by electrophoresis using a 1.0% agarose gel (Sangon Biotech Co), the product recovered by gel recovery kit (Sangon Biotech Co) and the recovered product used to add adenine at the end, according to the manufacturer's instructions. The amplified products were purified and cloned into pMD‐19T. The cloned products were then sequenced (Sangon Biotech Co). For each of the amplified fragments, three clones were sequenced.

The sequencing results were assembled by Geneious V4.8.4 software and the flanking sequence removed by sequence matching. The complete S gene was obtained and submitted to the NCBI database.

### 2.4 Phylogenetic analysis

Sequences of PEDV S gene Sichuan isolates were compared with other representative PEDV S gene sequences using MEGA V7.0 software. The phylogenetic tree was calculated using the neighbour‐joining (NJ) method. Bootstrap values were calculated based on 1,000 repeats of the alignment. The phylogenetic tree was used to analyse the genogroup evolution of PEDV isolates in Sichuan Province from 2018.

The amino acid/nucleotide sequence homology of the S gene of 25 strains and the amino acid differences between isolates and CV777 and CH/ZMDZY/11 were analysed by Clustal W with MegAlign software.

## 3 RESULTS

### 3.1 PEDV S gene amplification sequence

**Figure 1:**
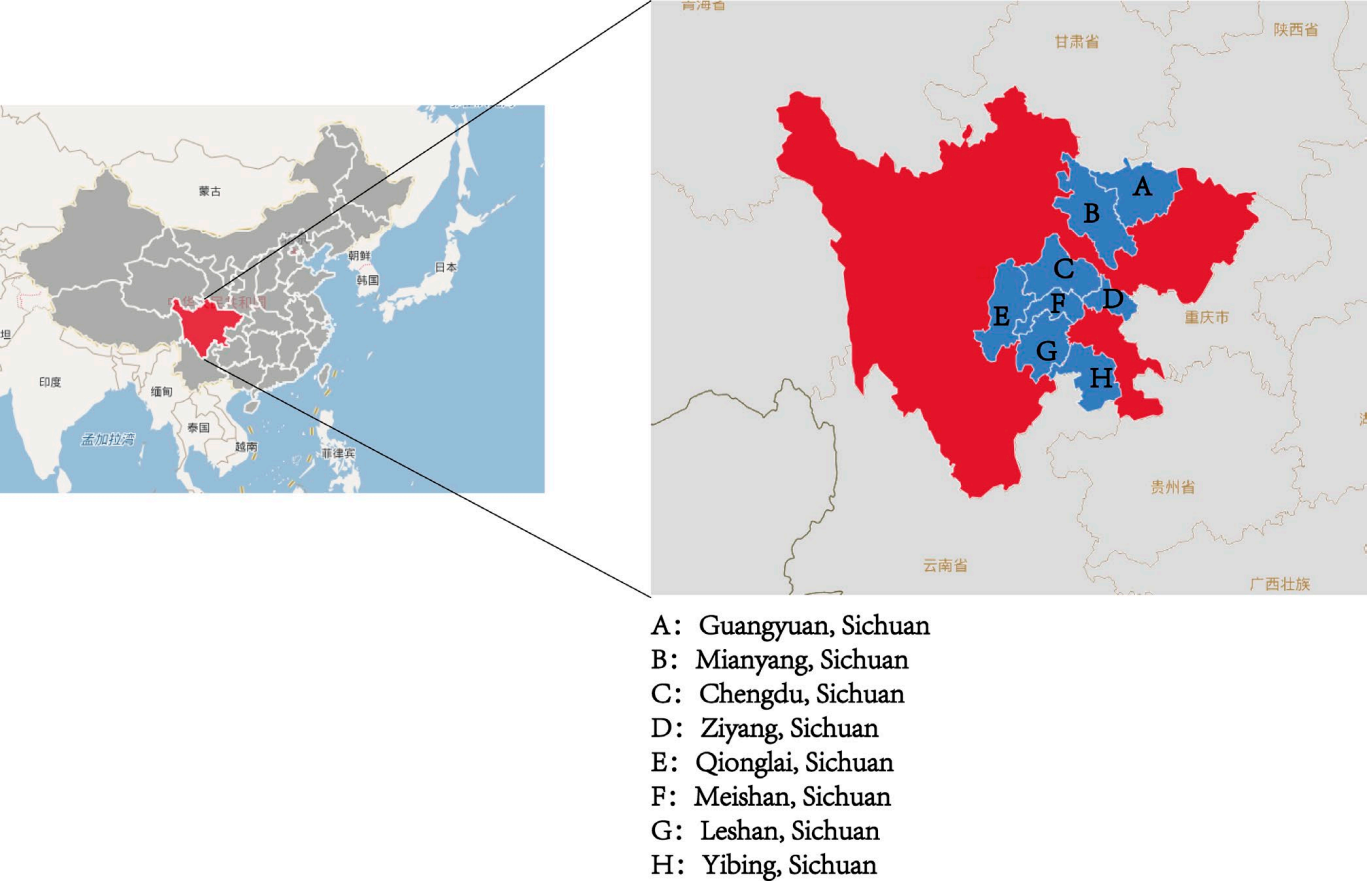
Distribution of isolates in Sichuan Province. Note: red: Sichuan Province, blue: Positive detection area

The clinical data of 10 PEDV samples and the size of S gene amplification fragments are shown in Table 2. Distribution of isolates in Sichuan Province is shown in Figure 1.

**Table 2:** Clinical sample information and amplified fragment size

Sequence number	Designation	Geographic origin	GenBank accession numbers	Genome size (bp)	Genogroup
1	CH/SCCD−1/2018	Chengdu, Sichuan	MN617858	4,161	GII‐a
2	CH/SCCD−2/2018	Chengdu, Sichuan	MN617859	4,161	GII‐a
3	CH/SCMS−1/2018	Meishan, Sichuan	MN617860	4,161	GII‐a
4	CH/SCZY−1/2018	Ziyang, Sichuan	MN617861	4,158	GII‐b
5	CH/SCMY−1/2018	Mianyang, Sichuan	MN617862	4,161	GII‐a
6	CH/SCGY−1/2018	Guangyuan, Sichuan	MN617863	4,152	GI‐a
7	CH/SCLS−1/2018	Leshan, Sichuan	MN617864	4,161	GII‐a
8	CH/SCYB−1/2018	Yibing, Sichuan	MN617865	4,161	GII‐a
9	CH/SCQL−1/2018	Qionglai, Sichuan	MN617866	4,161	GII‐a
10	CH/SCQL−2/2018	Qionglai, Sichuan	MN617867	4,161	GII‐a

### 3.2 Phylogenetic analysis of the S gene

**Figure 2:**
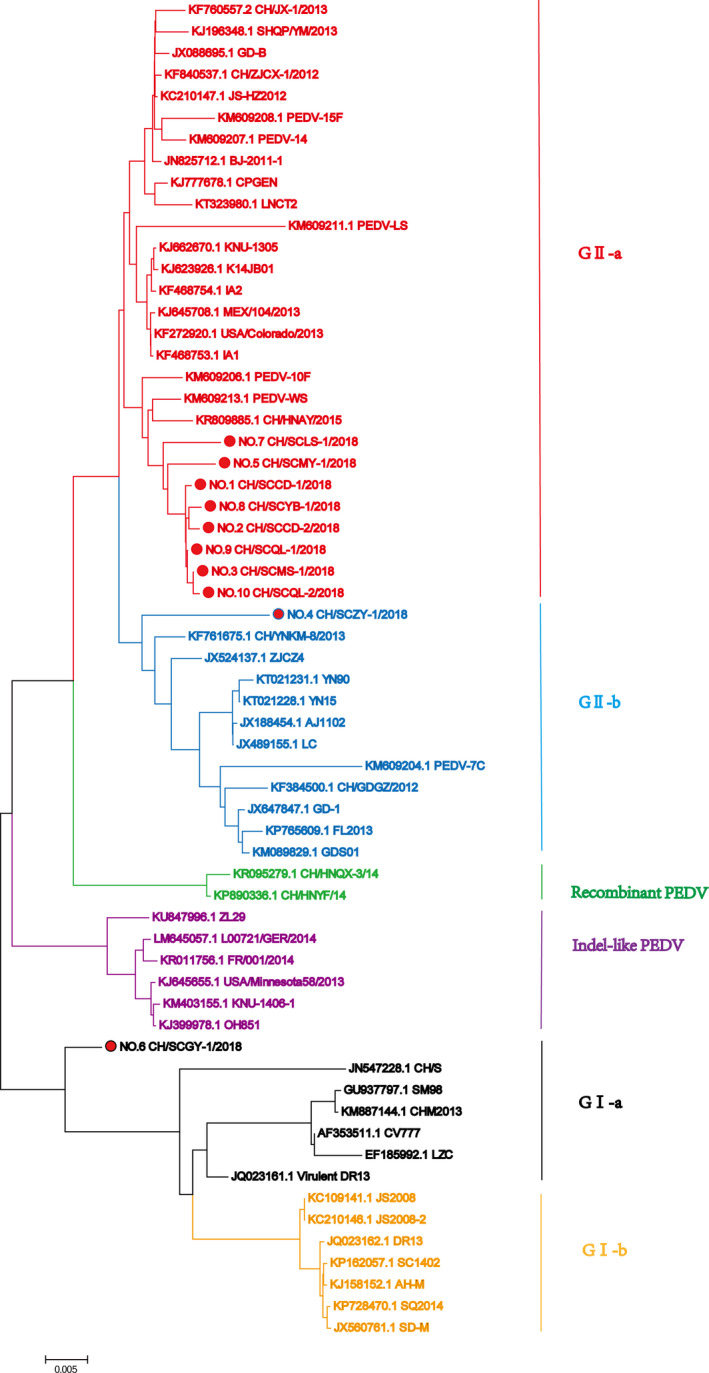
Phylogenetic tree analysis of the PEDV S gene

According to the phylogenetic analysis of the S gene, 9 of the 10 PEDV strains in this study were subtype GII and one was of subtype GI, distributed in three subgroups: GII‐a, GII‐b and GI‐a (Figure 2). In terms of the number of infections, GII‐a has become the main genogroup in Sichuan. Our GII subtype isolates showed a close relationship to CH/HNAY/2015, PEDV‐WS and PEDV‐10F isolates. Only one GI subtype isolate was closely related to CV777, CH/S and LZC isolates.

### 3.3 Alignment and analysis of amino acid sequence of the S gene

**Figure 3:**
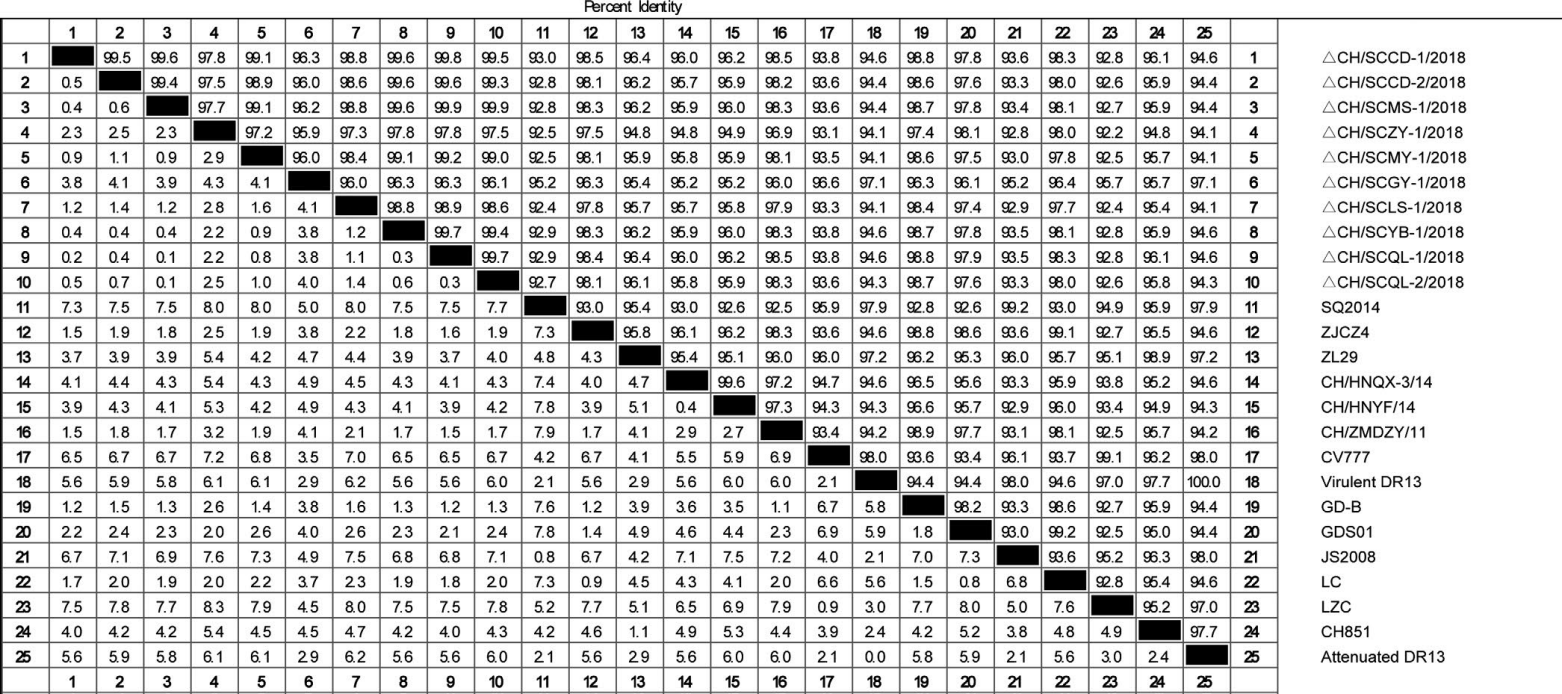
Amino acid sequence homology analysis of S gene of 10 isolates and 15 representative strains

**Figure 4:**
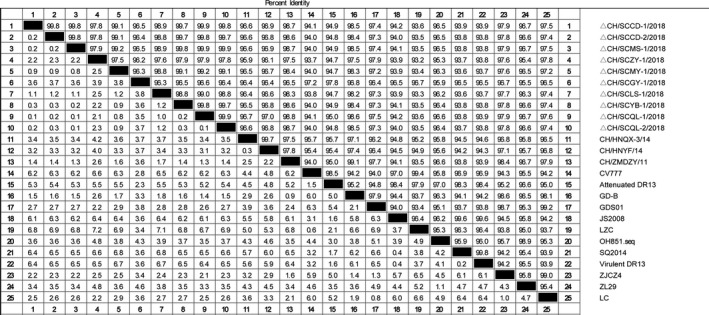
Nucleotide sequence homology analysis of the S gene of 10 isolates and 15 representative strains

The homology of amino acid sequence S of 10 isolates and 15 reference strains was analysed by MegAlign software in DNAStar. The results showed that the homology of amino acid sequence between isolates was 96.0%–99.9%, that of reference strains was 92.2%–98.8% and the amino acid homology between the isolate and CV777 was 93.1%–96.6%. (Figure 3). The nucleotide sequence homology of the S gene of the isolated strain was 96.2%–99.9%, that of the reference strain was 93.2%–98.8% and the nucleotide homology between the isolate and CV777 was 93.7%–97.2%. (Figure 4).

**Figure 5:**
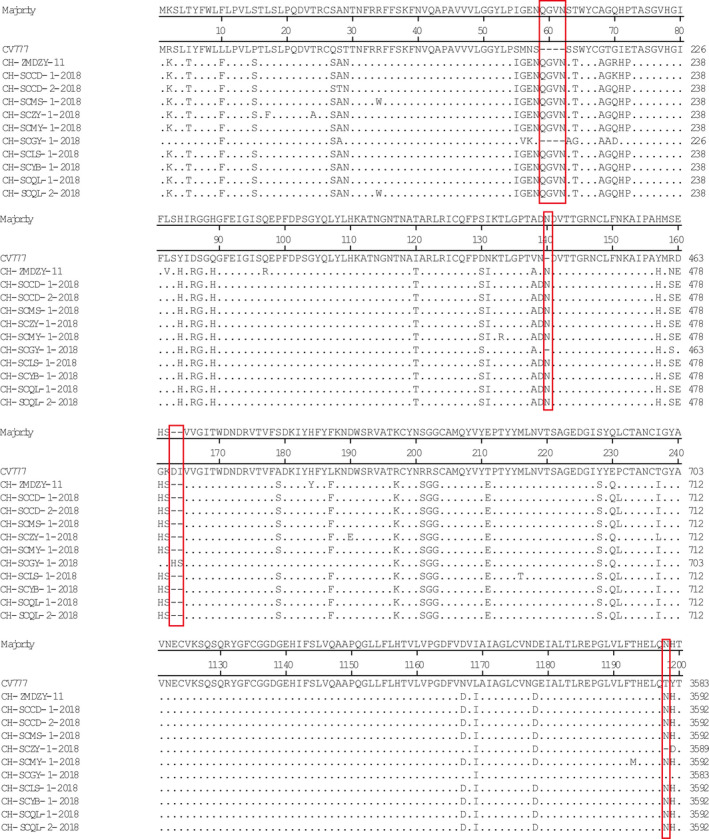
Analysis of amino acids encoded by the S gene of PEDV (different genogroup amino acid‐specific mutation sites are indicated in the box)

Compared with the CV777, except for CH/SCGY‐1/2018, the protein encoded by the amplified S gene had a four amino acids (QGVN) insertion at positions 58–59, a one‐amino acid (N) insertion at positions 135–136, a two amino acids (DI) deletion at positions 163–164 and a one amino acid (T) deletion at position 1,196 of CH/SCZY‐1/2018 (Figure 5).

We also examined the four major epitope regions, viz: COE (499–638aa), SS2 (748–755aa), SS6 (764–771aa) and 2C10 (1368–1374aa). The neutralizing epitopes of amplified S gene fragments were compared with the CV777. The results showed that the sequences at SS2 (748–755aa) and 2C10 (1368–1374aa) were conserved between the latest Sichuan PEDV isolates and CV777 strain, however, the sequences at positions COE (499–638aa) and SS6 (764–771aa) were variable (Table 3).

**Table 3:** Analysis of amino acid mutations in epitopes domains of field strains and the CV777 strain (499–638aa, 748–755aa, 764–771aa and 1368–1374aa located in CV777)

strains	507	516	517	520	521	523	527	536	549	594	605	609	612	621	633	635	766	768
CV777	H	A	A	G	L	S	V	F	T	G	A	G	L	K	E	I	Y	G
CH/ZMDZY/11			S		R	G	T		S	S	E		F			V		
CH/SCCD−1/2018			S		H	G	I	L	S	S	E		F			V	S	
CH/SCCD−2/2018			S	D	H	G	I	L	S	S	E		F			V	S	D
CH/SCMS−1/2018			S		H	G	I	L	S	S	E		F		V	V	S	
CH/SCZY−1/2018					H	G	I			S	D		F			V	S	
CH/SCMY−1/2018			S			G	I	L	S	S	E	V	F			V	S	
CH/SCGY−1/2018						G							F			V		
CH/SCLS−1/2018	R	G	G		H	G		L	S	S	D		F	Q		V	F	
CH/SCYB−1/2018			S	D	H	G	I	L	S	S	E		F			V	S	
CH/SCQL−1/2018			S		H	G	I	L	S	S	E		F			V	S	
CH/SCQL−2/2018			S		H	G	I	L	S	S	E		F		V	V	S	

## 4 DISCUSSION

At present, PEDV remains prevalent in China with several reports implicating the PEDV GII genogroup as the specific causative agent in many recent outbreaks (Daesub & Bongkyun, 2012; Douglas et al., 2013). Using the DNA sequence of the S gene fragment, we analysed the genetic variation and phylogenesis of PEDV strains collected in 2018 from farms in various regions of Sichuan Province. Our results revealed that eight of the amplified S genes were closely related to the isolates of the GII‐a genogroup, which were previously identified after the 2011 outbreaks in China and the United States. Moreover, our phylogenetic analysis showed that the PEDV strains in Sichuan Province were very similar to those previously reported (Wang, Fang, Fang, & Xiao, 2016), indicating that the currently prevalent PEDV strains in Sichuan are of GII genogroup.

In terms of PEDV vaccination, the current attenuated vaccines produced using the classical strains CV777 and ZJ08, which both belong to the GI genogroup, may not be sufficient to confer complete protection against the emerging PEDV strains of GII genogroup (Daesub & Bongkyun, 2012; Douglas et al., 2013). This partial immunoprotection has been demonstrated in clinical practice where pigs vaccinated against the classical strains (GI genogroup) remained vulnerable to PEDV infection (Luo et al., 2012; Sun et al., 2012; Tian et al., 2013). Researchers from China and the United States have also confirmed that all available commercial vaccines (GI genogroup) do not provide adequate immune protection against the currently prevalent strains (GII genogroup) (Lin et al., 2016; Opriessnig et al., 2017). After PED outbreaks in the United States in 2013, an inactivated vaccine was developed by Collin et al. (2015) based on the isolated US PEDV strain (GII genogroup) and was used to vaccinate a group of 4‐week‐old piglets. The inactivated vaccine was shown to trigger sufficient humoral immunity against PEDV thereby preventing subsequent infections (Collin et al., 2015). Baek et al. (2016) also developed an inactivated vaccine based on the Korean PEDV epidemic strain called the KNU‐141112. The administration of KNU‐141112–based vaccine to sows greatly increased the survival rate of piglets challenged with the virulent strain, and significantly reduced the severity of diarrhoea thereby minimizing the foecal shedding of the virus (Baek et al., 2016). Considering the differences between commercially available vaccines (GI genogroup) and the field‐epidemic strains (GII genogroup), China is now developing a vaccine based on AJ1102 (GII genogroup) isolates, which clinical re‐examination is underway (Wang, Fang, et al., 2016).

The S gene might correlate with PEDV pathogenicity (Martelli et al., 2008), implying that the S protein could be a potential vaccine candidate. Indeed, the S1 domain of the S protein is the major target for PEDV vaccine development (Oh, Lee, Lee, Choi, & Lee, 2014). Chinese researchers speculate that the amino acid mutations in the neutralizing epitope regions may be associated with the alterations in PEDV antigenicity, which may explain why the GI genogroup‐based vaccine cannot efficiently protect against infections caused by the GII genogroup or field strain (Li et al., 2014). Within the S protein, a series of single amino acid (aa) substitutions were found as indicated in the following positions: 517aa, 523aa, 549aa, 594aa, 605aa, 612aa and 635aa. These changes were observed in the strain CH/ZMDZY/11, which was previously isolated in central China (Xiao‐Meng et al., 2013). However, as shown in Table 3, there were other single aa mutations, suggesting that the gene encoding the antigenic domain of S1 may constantly vary across field isolates. In comparison with the CV777 strain, we found that, except for CH/SCGY‐1/2018 strain, the protein encoded by the S gene had four amino acids (QGVN) insertion at positions 58 to 59, one amino acid (N) insertion at positions 135 to 136 and two amino acids (DI) deletion at positions 163 to 164. In CH/SCZY‐1/2018 strain, we observed one amino acid (T) deletion at position 1,196. These deletions or additions are consistent with those of the Chinese isolate CH/ZMDZY/11 (Xiao‐Meng et al., 2013). Previous reports showed that the GI genogroup may have a weaker sugar‐binding activity than that of the field isolates (GII genogroup), and that there are differences in S protein antigens between the GI and GII genogroups. Moreover, it is speculated that the CV777 and other epidemic strains may show differences in their ability to enter the cells and induce fusion thereby causing a different effect on cell morphology (Deng et al., 2016; Wang, Chen, et al., 2016). Whether or not these changes affect the biological functions of PEDV will require further investigation. Furthermore, phylogenetic analysis of S genes showed that our field strains were highly similar to the reference strains (GII genogroup) but different from the CV777 strain, implying that the prevailing field strains in Sichuan province belong to the GII genogroup. Altogether, our results could provide reference data for future PED outbreaks and information for prospective prevention and control strategies, including vaccination and epidemiological predictions.

## CONFLICT OF INTERESTS

The authors declare that there is no conflict of interest.

## AUTHOR CONTRIBUTION

Fei Li: Investigation; Methodology; Writing‐original draft. Yubing Zeng: Investigation; Validation. Rubo Zhang: Resources. Kenan Peng: Investigation. Chaoyuan Jiang: Resources. Zhiwen Xu: Conceptualization; Supervision; Writing‐review & editing. Ling Zhu: Conceptualization.

## ETHICAL STATEMENT

The authors confirm that the ethical policies of the journal, as noted on the journal's author guidelines page, have been adhered to and the appropriate ethical review committee approval has been received.

### Peer Review

The peer review history for this article is available at https://publons.com/publon/10.1002/vms3.326.
